# Magnetic Field-Driven Transport Properties of an Oxygen-Deficient Rectangular YBa_2_Cu_3_O_7-δ_ Superconducting Structure

**DOI:** 10.3390/ma18163890

**Published:** 2025-08-20

**Authors:** Artūras Jukna

**Affiliations:** Photovoltaic Technologies Laboratory, Department of Physics, Faculty of Fundamental Sciences, Vilnius Gediminas Technical University (Vilnius Tech), Saulėtekio av. 3, LT-10257 Vilnius, Lithuania; arturas.jukna@vilniustech.lt; Tel.: +370-5-251-2462

**Keywords:** YBa_2_Cu_3_O_7-δ_ superconductor, partially deoxygenated superconducting structure, current–voltage characteristics, voltage steps, thermally activated flux flow, flux creep

## Abstract

The transport properties of biased type II superconductors are strongly influenced by external magnetic fields, which play a crucial role in optimizing the stability and performance of low-noise superconducting electronic devices. A major challenge is the stochastic behavior of Abrikosov vortices, which emerge in the mixed state and lead to energy dissipation through their nucleation, motion, and annihilation. Uncontrolled vortex dynamics can introduce electronic noise in low-power systems and trigger thermal breakdown in high-power applications. This study examines the effect of a perpendicular external magnetic field on vortex pinning in biased YBa_2_Cu_3_O_7-δ_ devices containing laser-written, rectangular-shaped, partially deoxygenated regions (δ ≈ 0.2). The results show that increasing the magnetic field amplitude induces an asymmetry in the concentration of vortices and antivortices, shifting the annihilation line toward a region of lower flux density and altering the flux pinning characteristics. Oxygen-deficient segments aligned parallel to the current flow act as barriers to vortex motion, enhancing the net pinning force by preventing vortex–antivortex pairs from reaching their annihilation zone. The current–voltage characteristics reveal periodic voltage steps corresponding to the onset and suppression of thermally activated flux flow and flux creep. These features indicate magnetic field–tunable transport behavior within a narrow range of temperatures from 0.94·*T*_c_ to 0.98*·T*_c_, where *T*_c_ is the critical temperature of the superconductor. These findings offer new insights into the design of vortex-motion-controlled superconducting electronics that utilize engineered pinning structures.

## 1. Introduction

The motion of Abrikosov magnetic vortices, as circular currents enclosing a magnetic field line that carries a single flux quantum Φ_0_ = *h*·(2*e*)^−1^ = 2.068 × 10^−15^ Wb, and antivortices, characterized by oppositely directed magnetic field lines, leads to energy dissipation in type II superconducting films biased with a supercritical current density *J*_c_. Here, *h* = 6.626 × 10^−34^ J⋅s is the Planck constant and *e* = 1.602 × 10^−19^ C is the electron charge [1,2]. The dissipation process manifests experimentally as a finite resistivity in the mixed state of the superconductor and can be divided into several stages: condensation of vortices/antivortices at opposite edges of the superconducting film at a magnetic field *H*_J_ ≥ *H*_c1_, their nucleation and motion toward the center of the film due to the action of the current’s self-produced Lorentz force, *F*_L_, and their annihilation at the center of the film with antivortices. Here, *H*_c1_ is the critical field at which magnetic flux begins to penetrate the superconductor in the form of Abrikosov vortices. At the moment of vortex–antivortex annihilation, a new pair is instantaneously generated. Under constant bias current conditions, corresponding to a fixed-current self-induced magnetic field *H*_J_, the number of vortex–antivortex pairs *n* remains constant and is described by the following relation [1]:

(1) $$n=\frac{\mu_{0}\cdot \mu \cdot H_{J}}{\Phi_{0}},$$

where μ_0_ = 4π × 10^−7^ H·m^−1^ is the magnetic permeability of free space, and μ is the effective magnetic permeability of the superconducting material in the mixed state.

**Figure 1:**
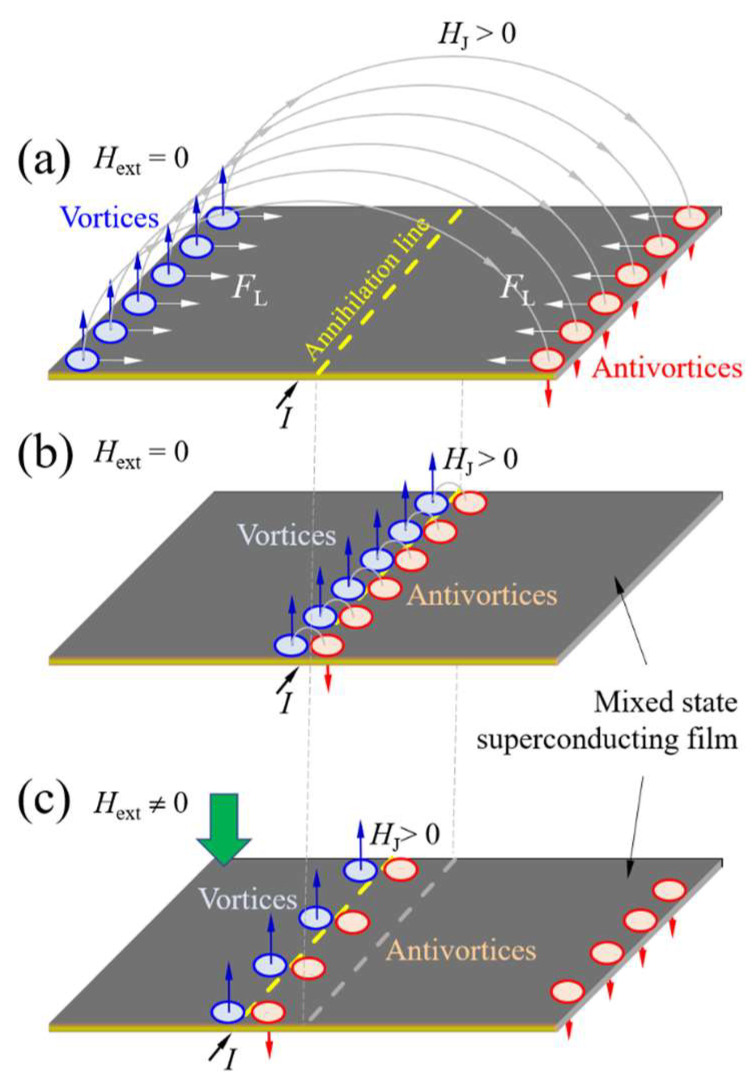
(**a**) When the pinning force *F*_p_ exceeds the Lorentz force *F*_L_, magnetic flux is unable to nucleate at the film edges. (**b**) Once *F*_L_ > *F*_p_, vortices and antivortices nucleate at opposite edges, move toward the center of the superconducting film, and annihilate there. (**c**) In the presence of an external magnetic field, the vortex–antivortex annihilation line shifts toward the side with a lower local magnetic flux density.

The Lorentz force, *F*_L_, arising from the self-induced crossed electric and magnetic fields due to the bias current, initiates the nucleation and subsequent acceleration of the magnetic flux (Figure 1). The rate of flux nucleation is determined by the amplitude of the pinning force, *F*_p_, originating from imperfections in the crystalline structure of the superconductor, e.g., one-, two-, and three-dimensional defects, such as impurities, inhomogeneities, and dislocations [3]. The resulting flux flow velocity, *v*, is inversely proportional to the effective viscosity η of the superconducting medium, which impedes vortex motion. This relationship is described [4] by:

(2) $$v=\frac{J\cdot \Phi_{0}}{\eta }\propto \frac{F_{L}-F_{p}}{\eta },$$

where *F*_p_ is lower than *F*_L_ for the flux flow to occur.

When the pinning force exceeds the Lorentz force (*F*_p_ > *F*_L_), the magnetic flux lines remain pinned and no vortex motion occurs (Figure 1a). The pinning force is directly related to the spatial variation in the vortex condensation energy [1,2,3,4,5]. These variations arise from differences between regions of weak and strong superconductivity [5], where the latter are typically characterized by a higher critical temperature *T*_c_, critical current density *J*_c_, and lower critical magnetic field *H*_c1_. Here, *T*_c_ is the maximum temperature at which the material remains superconducting, while *J*_c_ denotes the maximum current density that a type II superconductor can carry without inducing vortex motion. Under such conditions, only isolated vortex nucleation may occur, corresponding to the regime of thermally activated flux flow (TAFF).

As the applied current increases, the density of magnetic flux lines increases, leading to enhanced mutual repulsion because of their associated magnetic fields. This interaction facilitates the nucleation of additional vortices and results in energy dissipation as the vortices begin to move through the superconducting material. This behavior is interpreted as an effective reduction in the pinning force, since the pinning now acts on vortex bundles rather than isolated vortices. The balance between the Lorentz force and the collective pinning force of these bundles governs their dynamics. The motion of vortex bundles manifests itself as a sudden increase in energy dissipation, marking the onset of the flux creep (FC) regime [2].

In wide thin films, vortices nucleate at random locations along the edges, and their velocity is governed by intrinsic pinning within irregularly formed propagation pathways throughout the film. These pathways can be artificially controlled by selectively modifying the pinning strength in terms of modulating the film thickness [6] or patterning multilayer structures [7], modulating the film composition, or introducing defects, such as oxygen vacancies in cuprate superconductors [8], etc. It was recently shown [7] that comparatively strong pinning centers can be introduced in YBaCuO_7-δ_ superconducting films (*T*_c_~92 K) using a laser-writing technique [9]. Light absorption by the YBa_2_Cu_3_O_7-δ_ (YBCO) superconducting film results in an increase in the temperature of the illuminated areas and outflow of oxygen, producing oxygen vacancies. The appearance of oxygen vacancies causes a decrease in the *T*_c_, *J*_c_, and *H*_c1_ of the superconductor [10]. If δ exceeds 0.6, a material with an orthorhombic crystalline structure becomes an insulator with a tetragonal crystalline structure [11] and does not superconduct. However, in sites characterized by δ < 0.6, the superconductor has superconducting properties [7,8], showing lower values of *T*_c_, *J*_c_, and *H*_c1_ compared to sites that are rich in oxygen.

By engineering narrow, oxygen-deficient rectangular structures spaced several tens of micrometers apart, additional intrinsic pinning sites for magnetic flux can be introduced [7]. The sections of the structure aligned parallel to the bias current inhibit the formation of a vortex–antivortex annihilation zone in the center of the film, thus simultaneously restricting vortex motion along the segments perpendicular to the current. At current densities *J* >> *J*_c_, vortices pinned along segments parallel to the current become unpinned and move individually along perpendicular sections (TAFF regime). As the current density increases, strong repulsion between vortices pinned at the edges of the parallel segments leads to the collective unpinning and motion of vortex bundles, resulting in energy dissipation characteristic of the FC regime. This vortex dynamic manifests as voltage steps in the current–voltage characteristics of superconducting thin-film devices, containing partially deoxygenated rectangular structures. These steps provide a useful means for studying vortex behavior under an external magnetic field *H*_ext_ applied perpendicular to the film surface.

The current study shows that the application of *H*_ext_ creates an asymmetry in vortex and antivortex distributions, shifting the annihilation line toward areas of lower flux density (Figure 1c). Oxygen-deficient segments, aligned with the direction of current flow, act as barriers to vortex motion and improve the net pinning force by preventing vortex–antivortex pairs from reaching their annihilation zone. The results obtained can help to understand the role of an external magnetic field in controlling the pinning force within YBCO thin films provided with laser-written, rectangular-shaped oxygen-deficient structures. Moreover, since these engineered features are intended to suppress undesirable vortex dynamics, they can compromise the stability and performance of superconducting devices based on type II superconductors.

## 2. Sample Technology and Measurement Setup

**Figure 2:**
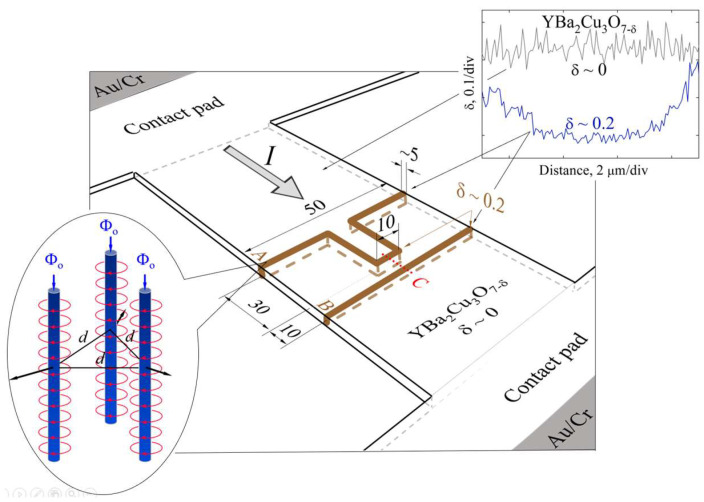
Schematic of the laser-patterned YBa_2_Cu_3_O_7-δ_ device (0.3 μm thick, 50 μm wide, and 100 μm long) with two 2 × 1.5 mm^2^ Au/Cr ohmic contact areas. Brown lines represent approximately 5 μm wide laser-written, oxygen-deficient regions (δ~0.2), aligned both parallel and perpendicular to the bias current. The inset on the right depicts the oxygen concentration profile in the deficient regions, highlighting the gradual slopes of oxygen depletion. The left inset shows that, under the experimental conditions, magnetic flux can penetrate the superconducting film in the form of Abrikosov magnetic vortices or antivortices, but exclusively in the brown regions.

Six YBCO devices, each with dimensions of 50 × 100 μm^2^, and two contact pads measuring 1.5 × 1.5 mm^2^ were laser-patterned in a nitrogen atmosphere from a 0.3 μm thick YBCO film. The film, with its *c*-axis oriented perpendicular to the substrate surface, was deposited on LaAlO_3_ substrates using a metalorganic chemical vapor deposition (MOCVD) method. Patterning was carried out using a 532 nm laser beam with an approximate diameter of 5 μ, operating at a power of 2.3–2.5 W and a scanning speed of 5 μm/s. Partial oxygen depletion (δ ≤ 0.2) in rectangular and straight-line structures (indicated by brown lines in Figure 2) was achieved in a subsequent laser-writing step using the same wavelength, but with a reduced power of approximately 0.4–0.6 W and an increased scanning speed of 50 μm/s.

As shown in Figure 2 (right inset), the laser-writing technique, which employs a Gaussian-shaped laser beam, produces sloped edges in the oxygen-depleted regions, creating a gradient in oxygen concentration. These sloped regions act as strong pinning centers, where Abrikosov vortices become trapped and interact magnetically with vortices traversing the 10 μm long central part of the rectangular structure [7]. Under the experimental conditions, magnetic flux penetrates the superconducting film exclusively in these regions (see Figure 2 (left inset)), forming Abrikosov magnetic vortices and antivortices. To more accurately assess the effect of laser modification on the electrical and magnetic properties, this study used a reference sample from the same YBCO film without laser-written structures and characterized it.

### 2.1. Technology of an Oxygen-Deficient Structure

When laser light interacts with the YBCO material in an oxygen-free ambient environment, it locally heats the illuminated areas and activates the diffusion of atomic oxygen from hotter to cooler areas. At higher laser powers (2.3–2.5 W), the oxygen content is significantly reduced, with the oxygen deficiency parameter reaching δ ≥ 0.6. Under these conditions, the temperature at the illuminated sites is estimated to reach or exceed 970 K, which corresponds to the orthorhombic to tetragonal phase transition of YBCO [11,12]. At such elevated temperatures and with a reduced oxygen content, the material becomes insulating and loses its superconducting properties. This degree of deoxygenation is achieved during the device patterning process, ensuring electrical isolation between devices and eliminating interference throughout the investigated temperature range.

At lower temperatures, corresponding to lower power (0.3–0.6 W), the diffusion of oxygen becomes significantly less pronounced. In YBCO crystals, oxygen diffusion occurs much more rapidly along the *a-b* planes than along the *c*-axis, by several orders of magnitude [13]. This anisotropy also affects the oxygen transport dynamics in the YBCO films used in this work, which were fabricated using the MOCVD method. In these films, the *c*-axis is oriented perpendicular to the substrate surface, effectively suppressing oxygen diffusion in that direction. Consequently, oxygen diffusion predominantly occurs parallel to the substrate, i.e., within the *a-b* planes. However, when nearby oxygen vacancy sites are fully occupied, atomic oxygen can only migrate toward the film surface, resulting in the formation of oxygen concentration gradients along the edges of the laser-modified regions. The steepness of these gradients also depends on the homogeneity of the film and the type and density of structural defects [13,14]. In particular, screw dislocations, commonly found in YBCO films grown by the MOCVD method, can improve local oxygen diffusion, potentially distorting the expected oxygen concentration gradient across the slope profile [13,14]. As a result, the efficiency of magnetic flux pinning at structure slopes cannot be accurately predicted by theoretical models alone, but must instead be evaluated experimentally by investigating vortex dynamics and their variation with an external magnetic field. Depending on its amplitude, the external magnetic field alters the density of flux lines, and, under a constant current (i.e., at a constant Lorentz force), can suppress flux flow in YBCO devices that contain oxygen-depleted structures.

The oxygen concentration profile in the laser-modified regions of the YBCO film (Figure 2, upper inset) presents the relative residual oxygen content as a function of distance within the optically modified structure (δ~0.2). The modification was produced in a nitrogen gas environment at room temperature using laser illumination with a power of approximately 0.5 W. The upper curve in the figure represents the residual oxygen concentration in a reference sample (δ~0). All measurements were made using the X-ray microanalysis technique. As previously reported by Jukna [7], the optically modified regions of the YBCO film exhibit characteristic oxygen concentration gradients that extend over a length of approximately 1 μm, which can serve as effective pinning sites for magnetic flux generated by electrical currents and external magnetic fields.

The described technology allowed the incorporation of two separate oxygen-deficient structures (δ~0.2) into 0.3 × 50 × 100 μm^3^ YBCO devices: a straight-line structure, 50 μm long and 5 μm wide (hereafter referred to as the linear structure), aligned perpendicularly to the current flow, and a rectangular-line structure (hereafter referred to as the rectangular structure), consisting of five 5 μm wide segments connected end-to-end. Two of these segments, each 30 μm long, are aligned parallel to the current flow (Figure 2). The central segment of the rectangular structure is positioned 10 μm away (center-to-center) from the linear structure.

### 2.2. Experimental Setup for Electrical and Magnetic Measurements

The experimental setup for electrical measurements consisted of a computer-controlled programmable current source (Keithley 2400 SourceMeter, Model Number 2400, Manufacturer: Keithley Instruments, Inc. Tektronix company, Cleveland, OH, USA) and a low-noise digital dc voltmeter (Keithley 2002 Multimeter, Model Number 2002, Manufacturer: Keithley Instruments, Inc. Tektronix company, Cleveland, OH, USA). The superconducting devices, current-biased during the measurements, were securely mounted on a copper cold finger inside a liquid nitrogen cryostat. The cryostat was operated under fore-vacuum conditions to prevent water vapor condensation and to ensure efficient thermal cooling.

The quality of electrical contacts was evaluated using square voltage pulses with durations of several tens of nanoseconds [15], confirming the ohmic behavior of the Au/Cr/YBCO interfaces at temperatures above *T*_c_. Four contacts for the standard four-probe measurement configuration (two inner electrodes used for voltage and two outer electrodes used for biasing) were fabricated by thermally evaporating gold onto chromium adhesion layers previously deposited on the YBCO film surface. These tests also demonstrated minimal contact heating under applied current [16], ensuring that the superconducting properties of the samples were not affected during measurements.

The critical temperature of the YBCO devices, both with and without partially oxygen-depleted structures, was determined from resistance versus temperature measurements performed using a standard four-probe configuration. A constant transport current of 10 μA was applied through the outer electrodes. The onset of the superconducting transition was defined as the temperature corresponding to the intersection point between two lines: one connecting 10% and 90% levels of resistive drop during the superconducting transition, and the other representing a linear extrapolation of the resistivity of the device in the normal (non-superconducting) state [17]. To eliminate the influence of thermoelectric voltages arising at metal/superconductor interfaces, measurements were conducted for both current polarities. The resulting voltage values were averaged to minimize the impact of thermoelectromotive effects at the interfaces.

The superconducting critical current *I*_c_ of YBCO devices was determined from the results of the current–voltage (*I-V*) characteristic. The critical current was defined as the dc current at which a voltage of 10 μV appeared across the inner voltage electrodes of the device. The critical current density was calculated as

(3) $$J_{c}=\frac{I_{c}}{W\cdot t},$$

where *W* = 50 μm denotes the width and *t* = 0.3 μm is the thickness, which were same for all tested YBCO devices, and *I*_c_ is the critical current of the superconductor. To eliminate residual magnetic memory effects, each measurement was preceded by a thermal cycling of the device above *T*_c_, followed by cooling to the target test temperature prior to current biasing.

A pair of Helmholtz coils produced a magnetic field perpendicular to the surface of the device. Each coil had a diameter of approximately 40 cm, and the pair was separated by about 20 cm to accommodate the cryostat. The sample, fixed to the copper cold finger inside the cryostat, was positioned near the midpoint between the coils, where the field is maximally homogeneous. A bias current flowing in the same direction through both coils generated a uniform magnetic field at the sample location, with the magnitude adjustable up to 13.3 mT. This configuration provided precise control of the magnetic field strength while maintaining uniformity across the superconducting device inside the cryostat. All measurements were made at temperatures below the *T*_c_ value of the superconductor. For repeated measurements, each new experiment was started only after the magnetic memory of the device was removed. This was achieved by heating the device slightly above its critical temperature *T*_c_, holding it at this temperature for several seconds, and then cooling it down to the desired experimental temperature below *T*_c_. In all experiments that involved the application of an external magnetic field, the field amplitude was gradually increased from zero to its maximum value.

## 3. Results and Discussion

### 3.1. Critical Current Density Under Zero and Applied Magnetic Fields

The as-prepared YBCO superconducting film exhibited a sharp superconducting transition within a narrow temperature interval of approximately 0.4 K, with the onset of the transition at *T*_c_ = 91.4 K. At liquid nitrogen temperature, the film demonstrated a high critical current density of approximately 3 MA/cm^2^. To ensure high measurement accuracy, this large critical current density was determined using square electrical pulses of nanosecond duration. The application of nanosecond pulses minimizes Joule heat at the contacts of the superconducting film in the mixed state [16] and allows accurate detection of the onset of a 10 μV voltage response induced by the transport current.

**Figure 3:**
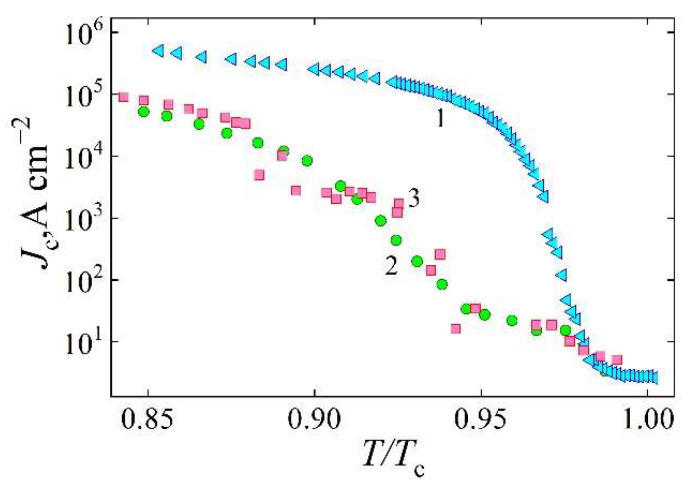
Dependence of the critical current density on the reduced temperature *T*/*T*_c_ for the reference sample (curve 1), the sample containing partially oxygen-decomposed structures in the absence of an external magnetic field (curve 2), and the same sample subjected to an applied magnetic field of 13.3 mT (curve 3).

Following the laser-patterning procedure, the superconducting properties of the reference sample showed noticeable degradation. Although the critical temperature remained nearly unchanged at approximately *T*_c_~91.4 K (the critical current density *J*_c_ was reduced to 0.21 MA/cm^2^ at a temperature of *T* = 78 K), the devices with laser-written rectangular and linear structures exhibited a significantly greater suppression of superconducting properties, showing a critical current density reduced by several orders of magnitude. Figure 3 presents the reduced temperature (*T*/*T*_c_) dependence of *J*_c_ in the range from 78 K to *T*_c_. The data are shown on a semilogarithmic scale for the following cases: the reference sample (curve 1), the sample containing partially oxygen-decomposed structures under a zero magnetic field (curve 2), and the same sample under an applied field of 13.3 mT.

At zero external magnetic field, the YBCO device containing partially oxygen-depleted structures exhibits two distinct steps in the critical current density as a function of reduced temperature, specifically in the intervals 0.94 < *T*/*T*_c_ < 0.98 and *T*/*T*_c_ < 0.9. These features are absent in the reference sample, indicating that the observed behavior is associated with magnetic flux flow within ten oxygen-deficient regions. Due to the reduced critical current density in these areas, the corresponding local lower critical magnetic field *H*_c1_ is also expected to be lower. As a result, these regions are more easily penetrated by the self-generated magnetic field to the transport current, leading to the formation of Abrikosov magnetic vortices. In contrast, the regions of the device not treated by laser retain significantly higher values of *H*_c1_, as reported in [18]:

(4) $$H_{c1}\;\sim \;\frac{\Phi_{0}}{4\pi \cdot \mu_{0}{\cdot \lambda }^{2}}\ln\frac{\lambda }{\xi }\;\sim \;J_{c}\cdot \lambda .$$

Here, *H*_c1_ denotes the lower critical magnetic field, corresponding to the onset of magnetic flux penetration into the superconductor, Φ_0_ is the magnetic flux quantum, λ is the London penetration depth, ξ is the coherence length of the YBCO superconductor, and *J*_c_ is the critical current density, characteristic of the material in the superconducting state. Since *J*_c_ in type II superconductors is limited by vortex penetration and the onset of vortex motion, Equation (4) leads to an approximation that allows the first critical field *H*_c1_ to be estimated as proportional to *J*_c_.

In contrast to the reference device, which does not contain oxygen-depleted structures and shows no significant response to external magnetic fields, the critical current density in the device with such structures is clearly influenced by the applied field. Curve 3 in Figure 3, corresponding to the measurements of the device with partially deoxygenated structures under an applied external magnetic field, generally follows the behavior of curve 2 (the zero-field case). However, a larger spread in the experimental *J*_c_ values is observed, indicating that the critical current density, associated with vortex motion in the oxygen-deficient regions, becomes less stable under the influence of an external magnetic field. Interestingly, in certain temperature intervals, the measured *J*_c_ values under the applied field exceed those under the zero field, while they are lower in others. This result suggests that the external magnetic field penetrates the oxygen-deficient regions and modifies vortex pinning, in some cases enhancing it and thereby increasing the critical current density. In particular, at reduced temperatures of *T*/*T*_c_ = 0.8735, 0.925, and 0.938, the critical current density increased by factors of approximately 1.8, 2.8, and 3.0, respectively (Figure 3).

The amplitude of the critical current density, as well as its dependence on temperature and magnetic field, is governed by the vortex dynamics [7]. An observed increase in *J*_c_ under a perpendicularly applied magnetic field of 13.3 mT indicates an enhancement of the pinning force *F*_p_, which impedes the motion of vortices and antivortices. This suppression leads to the onset of a superconducting state within the rectangular structure of the device provided with the rectangular structure of the optically modified material. The transition to the superconducting state is evident in the stepped *I-V* characteristics, where the dynamic resistance *dV*/*dI* shows distinct behavior. At temperatures *T* < *T*_c_, as demonstrated in [7], the *dV*/*dI* of the rectangular structure periodically becomes negligibly small or significantly large. The low-resistance states arise from the effective pinning of most vortices and antivortices, which suppresses the random nucleation and collective motion of vortex bundles, allowing only individual flux units to nucleate and move via thermally activated flux flow (TAFF). In contrast, the high-resistance states correspond to flux creep, which involves the collective motion of vortex bundles [2,7].

The applied magnetic field enhances the pinning force through several mechanisms. First, it induces an imbalance in the concentration of vortices and antivortices, leading to weaker compression between flux lines in the region of their lower density. As a result of the reduced repulsive interaction, the pinning of the vortex bundles becomes more effective. Although this effect is almost negligible in a linear structure (Figure 2) with an approximately uniform pinning force, it significantly influences pinning in a rectangular structure, particularly due to the presence of segments aligned parallel to the current flow.

Second, the applied field increases the magnetic interaction, known as the magnetic friction force [19], between stationary and mobile magnetic flux lines within the central segment of the rectangular structure. Due to the presence of segments aligned parallel to the current flow, the highest current density under a zero external magnetic field is concentrated in the central part of the device. This promotes vortex motion and annihilation in the central segment, which is oriented perpendicular to the current flow (Figure 2) [20]. These effects are further supported and analyzed through the stepped *I-V* characteristics observed in devices exposed to an external magnetic field. A detailed discussion of this analysis is presented in the following section.

### 3.2. Current–Voltage Characteristics

**Figure 4:**
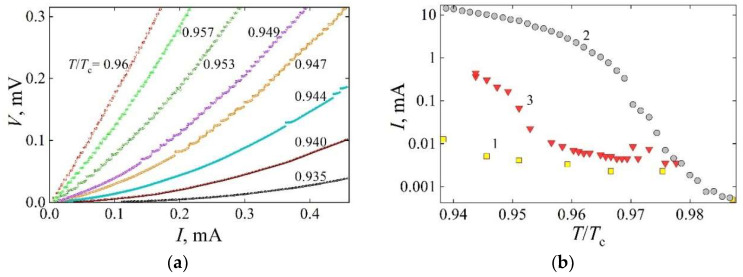
(**a**) Current–voltage characteristics of the YBCO device with rectangular and linear structures at various reduced temperatures ranging from 0.935·*T*_c_ to 0.96·*T*_c_. (**b**) Current corresponding to the onset of the first voltage step in the current–voltage characteristics of the same device, measured within the same temperature range (curve 3). Curves 1 and 2 represent the critical current of the same device and a reference device, respectively.

Nonlinear current–voltage characteristics of the 0.3 × 50 × 100 μm^3^ YBCO devices (Figure 4a), featuring rectangular and linear structures, exhibited voltage steps, observed in a narrow temperature range ranging from 0.94·*T*_c_ to 0.98·*T*_c_. Here, *T*_c_ denotes the superconducting transition onset temperature of the reference device and of the regions not treated by laser in the device containing oxygen-depleted structures.

As demonstrated by Jukna [7], there are no such voltage steps in the *I-V* dependences of the reference sample in the entire range of temperatures tested. As demonstrated in Figure 4b, the appearance of the first voltage step with decreasing temperature is dependent on the current amplitude, which is proportional to the amplitude of the current self-produced Lorentz force *F*_L_ acting on the current self-produced magnetic field, which penetrates the structures, and, due to oxygen vacancies, exhibit weak superconductivity, i.e., the lowest values of *T*_c_, *J*_c_, and *H*_c1_.

The nonlinear *I-V* characteristics indicate that the resistance of the device containing partially oxygen-decomposed structures increases monotonically with increasing current. At elevated temperatures, such as 0.96·*T*_c_, this dependence becomes nearly linear. Although the Δ*V*/Δ*I* of the device increases by approximately a factor of two at a bias current of 0.17 mA, the *I-V* curve exhibits a step-like behavior, beginning with the first voltage step at 4 μA and continuing beyond the 120th step at 2 mA (the upper limit selected to prevent device overheating). This behavior can be attributed to energy dissipation associated with vortex dynamics, primarily within the linear structure of the device. Specifically, the increasing self-induced magnetic field *H*_J_ (Equation (1)) leads to the condensation of additional vortex–antivortex pairs, while the enhanced Lorentz force *F*_L_ increases their velocity (Equation (2)) as they propagate toward the annihilation line. The linear structure, characterized by the presence of oxygen vacancies, exhibits nearly uniform pinning, which facilitates this dynamic behavior as the current increases, providing no reason for the appearance of voltage steps on the *I-V* curve.

The stepped-like behavior is attributed to vortex dynamics within the rectangular structure, where flux motion is constrained by the geometry, specifically, by additional pinning produced by segments aligned parallel to the current direction. Vortex lines tend to accumulate at the pinning centers formed along the sloped edges of these segments. They nucleate only when the self-induced Lorentz force exceeds the pinning force in the central segment, located between the two segments aligned parallel to the current (Figure 2). In these parallel segments, the Lorentz force directs the magnetic flux toward the sloped regions, where a gradient in the oxygen vacancy concentration enhances the pinning. These regions exhibit a pinning force much stronger than that elsewhere in the central region of the structure.

At higher temperatures of *T* ≈ *T*_c_, the onset of the first step in the *I-V* curves (Figure 4b) generally follows the 10 μV criterion, which is commonly used to determine the critical current of YBCO devices. However, at lower temperatures of *T* < 0.955·*T*_c_, such steps emerge only at significantly higher currents, exceeding the *I*_c_ by several orders of magnitude. This behavior suggests that the pinning force in the rectangular structure exceeds the Lorentz force, preventing the motion and annihilation of vortex–antivortex pairs. In contrast, such pairs can move and annihilate more easily in the linear structure. Since the pinning force increases with decreasing temperature [2,21], the current corresponding to the onset of the first step observed in the *I-V* characteristic also increases as the device temperature decreases. This behavior indicates that the step-like features of the *I-V* characteristic can be modulated by controlling the device temperature, which may be advantageous for practical applications of superconducting devices in both low- and high-power electronics. The response of the step-like *I-V* behavior to external magnetic fields in devices with partially deoxygenated structures is discussed in the following section.

### 3.3. Current–Voltage Characteristics and Voltage Steps Under an Applied Magnetic Field

**Figure 5:**
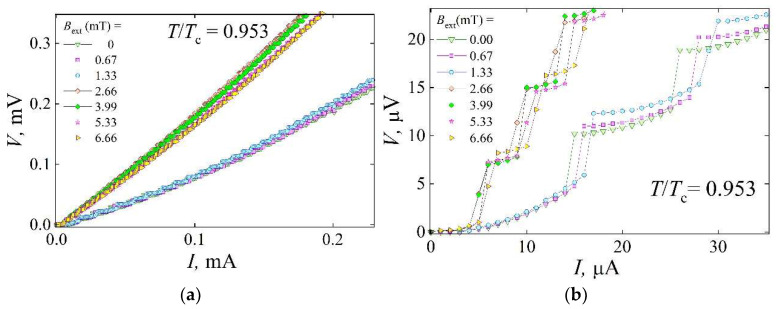
(**a**) Current–voltage characteristics of the YBCO device with rectangular and linear structures, measured at a fixed reduced temperature of 0.935·*T*_c_ under an external magnetic field ranging from 0 to 6.66 mT. (**b**) The same current–voltage characteristics shown on an expanded scale.

Since the vortex dynamics in the rectangular structure of the YBCO device govern the emergence of voltage steps in the *I-V* characteristics, these steps can serve as a key experimental indicator of the pinning properties of the sample. This phenomenon is attributed to the pinned vortex bundles or the vortex lattice [22] in the sloped regions of the structure segments aligned parallel to the current, which exhibit a gradient in the concentration of oxygen vacancies. The step-like *I-V* characteristics shown in Figure 5a were measured at a fixed temperature of 0.953·*T*_c_ under an applied perpendicular magnetic field. In particular, the voltage steps persist both in the absence and in the presence of an external magnetic field, with more than 100 steps observed in the range of currents *I* < 2 mA at 0 ≤ *H*_ext_ ≤ 6.66 mT. The same *I-V* curves, presented on an expanded scale in Figure 5b, illustrate the influence of the magnetic field on the overall *I-V* shape and on the morphology of the voltage steps.

As the current amplitude increases, the resulting Lorentz force initiates the motion of individual vortices within the rectangular structure, leading to energy dissipation that manifests as resistance in the mixed state of the superconductor. As shown in Figure 5a, the resistance increases with the increasing strength of the bias current and magnetic field. Furthermore, at a fixed temperature of *T* = 0.953·*T*_c_, when the external field *H*_ext_ exceeds 1.33 mT, the resistance exhibits an abrupt increase, approximately doubling compared to the values measured at *H*_ext_ < 1.33 mT. This change in resistance is attributed to a displacement of the vortex–antivortex annihilation line within the rectangular structure, shifting from the center toward the side with a lower concentration of magnetic flux lines. This shift results from the superposition of the current self-produced field and the external applied field. At *H*_ext_ > 1.33 mT, the vortex dynamics in the rectangular structure become similar to those observed in the linear structure of the device. As shown in Figure 5b, when the applied magnetic field exceeds 1.33 mT, the resulting imbalance in the concentration of vortices and antivortices causes the vortex dynamics to become less regular. This leads to a reduction in the current for the onset and development of the TAFF, while also destabilizing the flux (FC) for flux lines in the rectangular structure of the device. These effects are reflected in the changes in the slopes of the curves that represent the TAFF and FC regimes of flux flow.

The TAFF regime, characterized by a bias current interval associated with low dynamic resistance (Figure 5b), becomes nearly three times narrower at *H*_ext_ ≥ 2.66 mT, i.e., when the vortex–antivortex annihilation line is displaced from the central segment of the rectangular structure. In contrast, the voltage interval corresponding to energy dissipation due to the motion of the vortex bundles (FC regime) broadens with an increasing external magnetic field (Figure 5b). This broadening is reflected in the *I-V* dependencies as a decrease in differential resistance from 0.615 Ω under the zero field to 0.582 Ω at *H*_ext_ = 6.66 mT, representing a reduction of approximately 5.5% per voltage step in the current range near *J*_c_. This change in the FC-related region of the *I-V* curve suggests that the magnetic field enhances the effective pinning force acting on the moving vortices and that the vortex bundles involved in motion comprise a smaller number of vortices (bottleneck effect). This behavior is attributed to magnetic interactions between vortices moving within the central region of the rectangular structure and those pinned at the slope of the linear structure, which is located approximately 10 μm away (center-to-center) from it.

The increase in vortex concentration resulting from a higher external magnetic field also affects vortex dynamics in the linear structure. Vortices pinned along the slopes of linear and rectangular structures reduce the effective cross-sectional area available for vortex motion, thus narrowing the pathway for mobile vortices. As a result, a higher critical current density, or the fulfillment of the 10 μV criterion used to define it (Figure 5b), can be achieved at *H*_ext_ = 6.66 mT, compared to lower field values in the interval of 2.66 to 5.33 mT. This observation demonstrates the potential for tuning the critical current density in YBCO devices, incorporating linear and rectangular segments of partially deoxygenated material.

The monotonic increase in the critical current of the YBCO device, observed at *H*_ext_ ≤ 1.33 mT and a temperature of *T* = 0.953·*T*_c_, is also associated with a narrowing of the pathway for flux flow, as compared to the *I-V* characteristic measured under the zero magnetic field (Figure 5b). An increase in the magnetic field amplitude from 0 to 0.67 mT and 1.33 mT results in a corresponding increase in the critical current density from *J*_c_ = 100.0 A/cm^2^ to 106.7 A/cm^2^ (an increase of 6.24%) or 113.3 A/cm^2^ (11.76%), respectively. When weak magnetic fields are applied to the device, the current interval corresponding to the TAFF regime expands from *I* = 10 μA under the zero field to 11 μA (a 9.1% increase) at *H*_ext_ = 1.33 mT. Similarly, the voltage interval associated with energy dissipation due to the motion of vortex bundles (FC regime) increases with the applied magnetic field from Δ*V* = 6.15 μV under the zero field to 6.25 μV and 6.4 μV at 0.67 mT and 1.33 mT, respectively. This behavior indicates a reduction in the steepness of the *I-V* curve in the regions related to the FC regime, which, similar to the effect observed under the *H*_ext_ = 6.66 mT field, is attributed to the narrowing of the cross-sectional area for the vortex flow in the partially deoxygenated structures of the device.

Additionally, these results support the conclusion that the abrupt change in the shape of the *I-V* characteristic observed within the magnetic field range of 1.33 mT < *H*_ext_ < 2.66 mT is related to the displacement of the vortex–antivortex annihilation line from the central part of the device (in the central segment of the rectangular structure) toward the side of the structure with the lower concentration of magnetic flux lines. Because vortices can move perpendicularly to the current, this shift can occur in the segment aligned perpendicularly to the current and positioned farther from the linear structure of the YBCO device. This observation also suggests that each 1.33 mT increment in the external magnetic field corresponds to a 5 μm displacement of the vortex–antivortex annihilation line, such that the full 10 μm length of the central segment corresponds to a total field change of 2.66 mT in the partially oxygen-depleted YBCO device.

## 4. Conclusions

The obtained results demonstrate that the influence of a dc external magnetic field on the superconducting properties of current-biased YBCO devices can be effectively investigated using partially oxygen-depleted structures, revealing the interplay between current self-produced and external magnetic fields. The presence of slope regions with a gradient of oxygen vacancies, along with rectangular structures containing segments aligned parallel to the current, significantly enhances the pinning force, resulting in a higher critical current density compared to devices with only linear structures. The applied perpendicular magnetic field increases the pinning force for vortex motion flux flow in linear and rectangular structures. This effect is clearly reflected in the step-like *I-V* characteristics, where voltage steps correspond to distinct regimes of vortex dynamics, i.e., TAFF and FC. The external field introduces an imbalance between vortex and antivortex densities, leading to the displacement of the vortex–antivortex annihilation line toward a region of lower flux density. In turn, this compresses the vortex lattice in the current-aligned segments of the rectangular structure and narrows the effective cross-sectional area for vortex motion. These changes manifest themselves in the *I-V* curves as increased energy dissipation, a reduced vortex bundle size, and slower vortex motion with increasing magnetic field strength.

Overall, the results demonstrate that combining device geometry, specifically introducing a partially oxygen-depleted rectangular structure, with controlled oxygen depletion and an applied external magnetic field provides an approach for tuning the superconducting properties of cuprate-based devices. This strategy enables control over vortex behavior, offering advantages for advanced electronic and optoelectronic applications. By introducing partially oxygen-depleted rectangular structures and applying external magnetic fields as macroscopic control parameters, it is possible to manipulate quantum objects such as magnetic flux, whose nucleation and movement manifest as the appearance and modulation of mixed-state resistivity. Controlling vortex motion in type II superconductors is critical for low-noise superconducting transformers, superconducting single-photon detectors (SSPDs) [23], superconducting qubits, flux-based memory elements, rapid single-flux quantum circuits, superconducting microwave resonators, and magnetic sensors [24]. This approach can open pathways for optimizing device performance by reducing dissipation, enhancing signal-to-noise ratios, and improving stability in superconducting electronics.

## Data Availability

The original contributions presented in this study are included in the article material. Further inquiries can be directed to the author.
